# The 2025 Outstanding Contributions to ISCB Award—Dr Lucia Peixoto

**DOI:** 10.1093/bioinformatics/btaf263

**Published:** 2025-07-15

**Authors:** Mallory L Wiper

**Affiliations:** The International Society for Computational Biology, Leesburg, Virginia, United States

**Figure:**
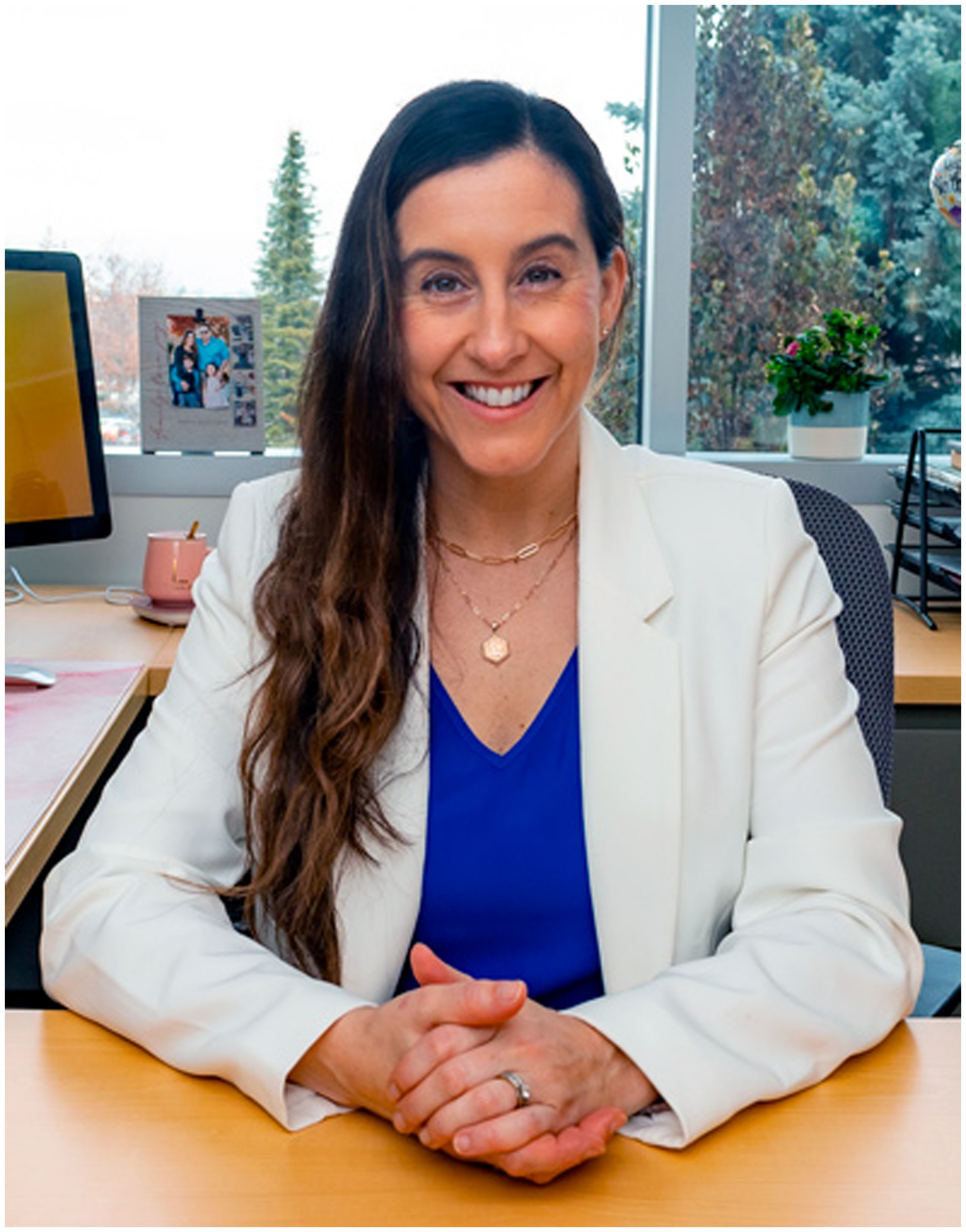


Every year, the International Society for Computational Biology (ISCB) presents the Outstanding Contributions to ISCB Award in recognition of a society member’s contributions to ISCB through their leadership, education, and service. This year, at the 33rd Annual Intelligent Systems for Molecular Biology (ISMB) conference and the 24th European Conference on Computational Biology (ECCB), ISCB is proud to present the Outstanding Contributions to ISCB award to Dr Lucia Peixoto!

## A journey into ISCB leadership

What began as a simple suggestion from a fellow PhD student would soon spark Peixoto’s long-standing leadership within ISCB. Prompted by that encouragement, Peixoto attended the 2007 Student Council Symposium (SCS), held ahead of the main ISMB conference in Vienna that year. Attending the symposium proved to be a turning point: not only did the SCS make the larger conference feel less intimidating, it also introduced her to a vibrant community of peers and marked the beginning of her long-standing involvement with ISCB.

At the close of the symposium, when the chairs, Nils Gallenburg and Manuel Corpas, asked for volunteers to help organize next year’s event, Peixoto raised her hand without hesitation. Her offer to volunteer quickly turned into a request for her to chair the 2008 symposium. Though she had never chaired a symposium before, she was up to the challenge.

This “say yes and figure it out later” approach was only the beginning of her leadership within ISCB. In 2009, she co-chaired the SCS symposium in Stockholm, and, that same year, became the SCS representative to the ISCB Board of Directors.

## Championing access and inclusion

While she enjoyed being part of ISCB and the SCS, she noted a distinct lack of diverse lived experiences within the community, particularly of scientists and students from underrepresented regions. This observation, in conjunction with her deep motivation to help people achieve their full potential regardless of background or location, played a crucial role in shaping her motivation to stay involved with ISCB.

Being born and raised in Uruguay, Peixoto knew firsthand how difficult access to an international conference could be and she wanted to change that. She wanted *everyone*, no matter where they were from, to have access to the same experiences and opportunities she’d had with ISCB. So, in her role as SCS representative to the ISCB Board of Directors, she helped organize the first ISCB Latin America conference in 2010, which took place in her hometown in Uruguay, to bring an international conference to the global south.

## A vision for ISCB’s future

When asked about the role she sees ISCB fulfilling to support the computational biology community in the future, Peixoto emphasized hope, strength, belonging, and the importance of ISCB as a supportive force. She sees ISCB standing as a source of strength and resilience, especially at a time when the value of science and scientific institutions are being tested. A strong advocate for data-driven decision-making, Peixoto hopes ISCB will continue to affirm that everyone belongs in computational biology and that all ideas are valued equally, recognizing that diversity of thought and background drives scientific excellence. More broadly, Peixoto highlighted that scientific societies have a responsibility to lead with clarity and conviction, ensuring that their actions reflect their values to help build a more inclusive future for the field.

## Service shaping science

Service, both within ISCB and beyond, has been a source of deep fulfillment as well as a source of inspiration for Peixoto’s research.

Within ISCB, Peixoto was the founding chair and is the current co-chair, of ISCB’s Equity, Diversity, and Inclusion committee. In this role, she continues to focus on bringing data-driven approaches to improving and promoting equity and inclusion in science, a responsibility about which she is very passionate.

Beyond ISCB, Peixoto’s research in neurodevelopmental disorders has been profoundly shaped by her engagement with patient communities. Her interactions with patients and their caregivers have been extraordinarily meaningful and have shaped her research in unexpected ways. A defining experience exemplifies how these interactions have shaped the direction of her research: Early in her career, Peixoto went to an open meeting on neurodevelopmental disabilities where parents, caregivers, and patient advocates were in attendance. She had been trying to decide on her research focus and while many papers were being published about the “hot” topics in the neurodevelopmental field, she thought, why not get the opinion of a non-scientist on what’s most important to study. When she posed this question to Geraldine Bliss, a parent at the meeting and the founder and president of cureSHANK (https://www.cureshank.org), the response was immediate: “sleep.” That simple, one-word answer shifted the entire trajectory of Lucia’s research, leading her to explore how sleep disturbances affect gene expression in the brain and their role in neurodevelopmental disorders. Her work continues to focus on the intersection of sleep, neurodevelopment, and autism, a path shaped by these community interactions.

Peixoto is also passionate about STEM outreach, which she conducts through her involvement in public school programs and the Girl Scouts of North America. She loves speaking to school-aged children about science and especially enjoys speaking to young girls, motivating them to pursue STEM careers.

Altogether, these experiences have reinforced a core belief for Peixoto: listening to and engaging with diverse communities, including non-scientists, is crucial to conducting meaningful, impactful research.

## Advice for the next generation

In speaking about what service opportunities she would recommend junior scientists or trainees seek out, Peixoto said that it’s important for people to pursue the opportunities that bring them joy because personally unrewarding service can quickly lead to burnout. She encourages younger generations to pursue a holistic experience in their scientific careers, noting, “I want to dispel the myth that doing service stands in opposition of doing great science.” To be a great scientist, Peixoto says, isn’t just about valuable research and scientific contributions; it’s also about being a great mentor and giving back to your community.

## Reflections on the Outstanding Contributions Award

Peixoto feels deeply honored to be the recipient of the 2025 Outstanding Contributions to ISCB award since ISCB has been such an integral part of her academic career.

She hopes that this award helps demonstrate to others in the field, no matter where they are in their career path, that science and service can co-exist, and she hopes, too, that this encourages more scientists to engage in service and that true excellence is about more than publishing papers.

